# Inflammatory changes underlying sepsis‐induced cognitive deficits in the Tau P301S mouse model of Alzheimer’s disease

**DOI:** 10.1002/alz.089875

**Published:** 2025-01-03

**Authors:** Camryn Smith, Adriana Tienda, Fiona E. Harrison

**Affiliations:** ^1^ Vanderbilt University, Nashville, TN USA; ^2^ Department of Medicine, Vanderbilt University Medical Center, Nashville, TN USA

## Abstract

**Background:**

The prevalence of sepsis and delirium in the elderly is a risk factor for subsequent diagnosis of Alzheimer’s disease and related dementias (ADRD). Post‐sepsis impairments include changes in memory, attention, emotional function, and neuromuscular strength. Studies have shown a link between the prolonged activation of microglia after infection. However, the exact mechanisms remain unclear. Challenges in modeling delirium in animal models include use of young animals, short trial endpoints, and sickness behaviors that can confound behavioral assessments. We sought to address these challenges using animals with additional ADRD‐related neuropathology. We hypothesized that age and ADRD neuropathology will be associated with greater and prolonged microglial dysfunction, and poorer behavioral outcomes after sepsis.

**Method:**

Male and female Tau P301S mice were used for this study at 5 months of age. These mice exhibit tau tangles, microgliosis and cell loss by 8 months of age. Mice of each genotype received either a polymicrobial cecal slurry injection to cause sepsis, or a vehicle control injection. Health scores of the mice were collected across the sickness timeline. Four days after infection, following recovery, behavior paradigms were conducted including y‐maze, fear conditioning, activity, rotarod, and nest‐building. The mice were then euthanized, and brains and plasma were collected to measure the progression of Alzheimer’s pathology and inflammatory response post‐infection.

**Result:**

Male mice of both genotypes were more sensitive to the cecal slurry treatments than females as indicated by sickness scores and time to recover. In male mice, P301S mice were more sensitive to the chronic effects of sepsis with greater changes in locomotor activity and tests of learning and memory.

**Conclusion:**

The data supports the link between poorer outcomes following sepsis in the presence of existing Alzheimer’s pathology and highlight a difference in the response to a systemic inflammatory challenge between male and female mice. These mice were tested prior to major development of ADRD‐related pathology to understand how additional inflammatory challenges may drive progression of ADRD pathology and behavioral changes. Future studies will confirm the range of neuropathological and neuroinflammatory changes in these mice.

